# A Bayesian network analysis quantifying risks versus benefits of the Pfizer COVID-19 vaccine in Australia

**DOI:** 10.1038/s41541-022-00517-6

**Published:** 2022-08-11

**Authors:** Jane E. Sinclair, Helen J. Mayfield, Kirsty R. Short, Samuel J. Brown, Rajesh Puranik, Kerrie Mengersen, John C. B. Litt, Colleen L. Lau

**Affiliations:** 1 School of Chemistry and Molecular Biosciences, Faculty of Science, The University of Queensland, Brisbane, QLD Australia; 2 School of Public Health, Faculty of Medicine, The University of Queensland, Brisbane, QLD Australia; 3 Department of Cardiology, Royal Prince Alfred Hospital, Sydney, NSW Australia; 4 Sydney Medical School, Faculty of Medicine and Health, The University of Sydney, Sydney, NSW Australia; 5 School of Mathematical Sciences, Faculty of Science, Queensland University of Technology, Brisbane, QLD Australia; 6 Discipline of General Practice, College of Medicine and Public Health, Flinders University, Adelaide, SA Australia; 7 Scientific Advisory Committee, Immunisation Coalition, Melbourne, VIC Australia

**Keywords:** Risk factors, Epidemiology, Epidemiology, Cardiovascular diseases, Viral infection

## Abstract

The Pfizer COVID-19 vaccine is associated with increased myocarditis incidence. Constantly evolving evidence regarding incidence and case fatality of COVID-19 and myocarditis related to infection or vaccination, creates challenges for risk-benefit analysis of vaccination. Challenges are complicated further by emerging evidence of waning vaccine effectiveness, and variable effectiveness against variants. Here, we build on previous work on the COVID-19 Risk Calculator (CoRiCal) by integrating Australian and international data to inform a Bayesian network that calculates probabilities of outcomes for the delta variant under different scenarios of Pfizer COVID-19 vaccine coverage, age groups (≥12 years), sex, community transmission intensity and vaccine effectiveness. The model estimates that in a population where 5% were unvaccinated, 5% had one dose, 60% had two doses and 30% had three doses, there was a substantially greater probability of developing (239–5847 times) and dying (1430–384,684 times) from COVID-19-related than vaccine-associated myocarditis (depending on age and sex). For one million people with this vaccine coverage, where transmission intensity was equivalent to 10% chance of infection over 2 months, 68,813 symptomatic COVID-19 cases and 981 deaths would be prevented, with 42 and 16 expected cases of vaccine-associated myocarditis in males and females, respectively. These results justify vaccination in all age groups as vaccine-associated myocarditis is generally mild in the young, and there is unequivocal evidence for reduced mortality from COVID-19 in older individuals. The model may be updated to include emerging best evidence, data pertinent to different countries or vaccines and other outcomes such as long COVID.

## Introduction

In December 2020, the Pfizer vaccine (BNT162b2; Cormirnaty) became the first COVID-19 vaccine to be authorised for public use^1^, and has since had >1.5 billion doses delivered to 131 countries^2,3^. In June 2021, reports linking the Pfizer vaccine to myocarditis, especially in male adolescents and young adults, started to emerge in Israel^4^ and the USA^5^. Despite low case numbers, this association informed government policies surrounding a slower vaccine rollout in younger age groups around the world^6^. Furthermore, intense media focus on this uncommon adverse event may have contributed to an increase in vaccine hesitancy in younger age groups^7^, especially in Australia where it was the only COVID-19 vaccine recommended for those aged under 60 years at the time^8^.

Having access to transparent information on the risks and benefits based on the current best available evidence is crucial for individuals to make an informed decision on whether to get vaccinated^9,10^, and also for informing public health policy regarding the recommendation of different vaccine types for different population subgroups. The Australian Technical Advisory Group on Immunisation (ATAGI) produced a helpful document on ‘Weighing up the potential benefits against risk of harm from COVID-19 vaccine AstraZeneca’^11^ to address concerns of vaccine-associated thrombosis with thrombocytopenia syndrome. While ATAGI released a clinical ‘Guidance on myocarditis and pericarditis after mRNA COVID-19 vaccines’^12^, there have not been any documents focused on risk-benefit analysis.

By October 2021, 23.4% and 55.1% of Australians aged over 16 years had received one and two doses of a COVID-19 vaccine, respectively, and an unspecified but small percentage had received a third dose^13^. Because of concerns related to the risk of thrombosis and thrombocytopenia syndrome with the AstraZeneca COVID-19 vaccine, the Pfizer vaccine was the standard recommendation for those aged < 60 years^14^. However, 6-month Pfizer vaccine effectiveness data that became available in October 2021 showed concerning reductions in protection against symptomatic infection each month after administration of the second dose^15^. In the context of the reopening of Australian borders in December 2021 and the introduction of the highly transmissible omicron variant, this decrease in vaccine effectiveness may leave even those who have had two doses of a COVID-19 vaccine at substantial risk of developing symptomatic COVID-19. Even for the highly vaccinated population of Australia, it was therefore crucial to communicate the necessity of third doses for maintaining optimal protection against symptomatic infection, serious illness and death.

To effectively facilitate this communication, a risk-benefit analysis tool capable of integrating best evidence from multiple data sources (both Australian and international) and formats (government reports, published literature and expert opinion) was required^16^. Furthermore, this tool must be easy to update as the pandemic landscape rapidly evolves and as more data become available. We have previously developed a Bayesian network (BN) model to analyse the risks and benefits of the COVID-19 AstraZeneca vaccine in the Australian population^17,18^. This model was used to programme the COVID-19 Risk Calculator (CoRiCal)^19^, a freely available user-friendly online tool that enables scenario analysis based on user inputs (age, sex, vaccination status, transmission scenario). The tool provides risk estimates for targeted subgroups and can be used by health managers as well as individuals alone or in conjunction with their general practitioner for shared decision-making on vaccination. This study describes the development of the BN model used to programme the second version of the CoRiCal tool, which focuses on the risks and benefits of the COVID-19 Pfizer vaccine for the Australian context, and details the results of the population-level risk-benefit analysis performed using this model, factoring in emerging evidence of waning vaccine effectiveness over time and the new recommendation for third doses.

## Results

### Model description

The BN model was designed to predict five outcomes:

i. Probability of developing and dying from Pfizer vaccine-associated myocarditis (n5, n12)—depending on vaccine dose (n1), age (n2) and sex (n3);
ii. Background probability of developing and dying from myocarditis (in those who have not had Pfizer vaccine or COVID-19) (n6, n13). Estimates were converted to probability of events over 2 months to enable comparison with the probability of vaccine-associated (n5, n12) and infection-associated outcomes (n10, n14) over 2-month periods;
iii. Probability of symptomatic COVID-19 (n10)—depending on intensity of community transmission (n4), vaccine effectiveness against symptomatic infection (n7), relative risk of symptomatic infection by age and sex (n9);
iv. Probability of dying from COVID-19 (n14)—depending on age (n2), sex (n3), vaccine effectiveness against death (n8); and
v. Probability of developing and dying from COVID-19-related myocarditis (n11, n15)—depending on age (n2), sex (n3).

**Fig. 1:**
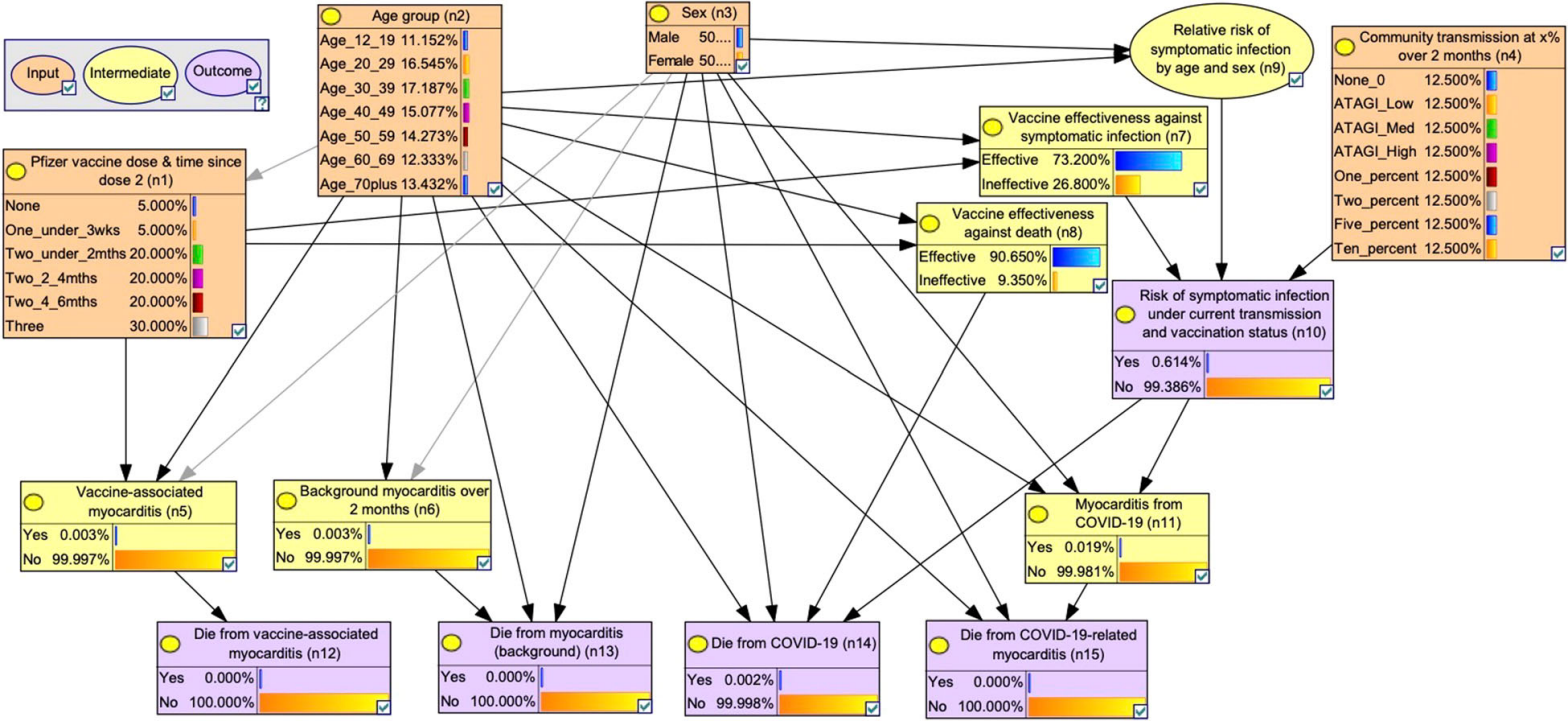
Bayesian network for assessing risks versus benefits of the Pfizer COVID-19 vaccine in Australia. Input nodes in orange (n1–n4), intermediate nodes in yellow (n5–9, n11), and outcome nodes in purple (n10, n12–15). All nodes are shown in their default states.

The BN (Fig. 1) displays the links between variables and outcomes based on the assumptions presented in Table 1^11,15,20–35^ and Supplementary Tables S1–9. Supplementary Table 10 summarises each of the 15 nodes and their parent/child associations. While some links are largely association-driven (e.g., the links between age and sex and relative risk of symptomatic infection), others are more causal (e.g., the link between developing COVID-19-related myocarditis and dying from it).

**Table 1:** Summary of data sources, assumptions and prior distributions for a Bayesian network to assess risks versus benefits of the Pfizer COVID-19 vaccine.

Model inputs	Data sources, assumptions, rationale (references)
Vaccine effectiveness against symptomatic infection	1 dose^20^ • Data from 503,875 individuals in Israel, 13 to 24 days after immunisation • Age < 60 years: 53.1% effective. Age ≥60 years 46.8% effective • Study conducted when delta was dominant variant. 2 doses^15^ • Data from large integrated health system in the USA • Data not specifically for delta variant but for a mix so we assumed there would be negligible difference between variants. • Our model focuses on risk of symptomatic infection, but this study reports estimates for total risk of infection (not necessarily symptomatic). Our model may therefore have underestimated vaccine effectiveness against symptomatic infection. • The study reports vaccine effectiveness at < 1 month, 1 to < 2 months, 2 to < 3 months, 3 to < 4 months, 4 to < 5 months and ≥5 months since the second dose. When transforming these data to the time categories used in our model (0 to < 2 months, 2 to < 4 months and 4 to < 6 months), we averaged the reported vaccine effectiveness of the respective months in each group. • In transforming the reported age groups to those used in our model, we assumed that in age group 12–19 years, 50% were aged 12–15 years and 50% were aged 16–19 years. Likewise for age group 40–49 years we assumed that 50% of people were aged 40–44 years and 50% were aged 45–49 years. Similar assumptions were used for 50–59 and 60–69 year-olds. • See Table S1 for summary of final assumptions. 3 doses^21^ • Data from Pfizer third dose efficacy study conducted in the USA, Brazil and South Africa • Prespecified analysis was performed 2 months after last participant enroled; blinded follow-up time after booster administration was < 2 months for 3% of the study population and ≥2 to < 4 months for 97% of the study population. • Age 16–55 years: 96.5% effective. Age ≥56 years: 93.1% effective • Study conducted when delta was the dominant variant. • We assumed vaccine effectiveness in ages 12–15 years was the same as in ages 16–55 years • In transforming reported age groups to those used in our model, we assumed that in age group 50–59 years, 60% were 50–55 years and 40% were 56–59 years. • See Table S1 for summary of final assumptions.
Vaccine effectiveness against death if infected	1 dose^22^ • Data from Ontario study, reporting vaccine effectiveness against hospitalisation or death from delta variant ≥12 days after first dose administration. These data may therefore underestimate effectiveness against death. • Age < 60 years: 89% effective. Age ≥60 years: 74% effective. 2 doses^23^ • Data from Public Health England reporting vaccine effectiveness against death from delta variant. • In transforming reported time since second dose into the categories used in our model, we used weighted averages of the vaccine effectiveness in different time groups reported in the study, with weighting being proportionate to the number of weeks in each category. • In transforming the reported age groups to the categories used in our model, we assumed that for age group 60–69 years, 50% were 60–64 years and 50% were 65–69 years. • Data were reported only for age groups ≥16 years (which includes ≥65 years) and ≥65 years. As data were not provided for ages 16–64 years only, we assumed estimates were the same as for the ≥16 years age group. It is therefore possible that vaccine effectiveness for this age group was underestimated due to influence of the lower effectiveness within the ≥65-year-olds. • As no data were reported for age < 16 years, we assumed that ages 12–15 years had the same vaccine effectiveness as ages 16–64 years. See Table S2 for summary of final assumptions. 3 doses^23^ • As no data have yet been published on third dose effectiveness against death, we assumed the same effectiveness as ‘Two doses (last dose 0 to < 2 months ago)’.
Relative risk of symptomatic infection by age and sex	Data from Australian National Interoperable Notifiable Diseases Surveillance System (NINDSS)^24^ reports age and sex distribution of all COVID-19 cases in Australia up to 8 Dec 2021. We subtracted data from the Australian Government Department of Health Epidemiology Reports 32 and 43^25^ reporting age and sex distribution of COVID-19 cases in Australia in 2020, and Jan to June 2021, respectively, to obtain age and sex distribution of cases from 6 June to 8 Dec 2021 to represent the delta variant. We calculated relative risk of infection by age group and sex by estimating the probability of infection in each age-sex group if overall probability of infection in the community was 1%. See Table S3 for final assumptions.
Risk of symptomatic infection under current transmission and vaccination status	Definitions of low, medium and high transmission as defined by Australian Technical Advisory Group on Immunisation (ATAGI)^11^. Low—similar to first wave in Australia (equivalent to 0.016% of population infected over 2 months). Medium—similar to second wave in Victoria, Australia in 2020 (equivalent to 0.149% of population infected over 2 months). High—similar to Europe in January 2021 (equivalent to 1.920% of population infected over 2 months). Also included transmission scenarios equivalent to: zero transmission; 1%, 2%, 5% and 10% chance of infection over 2 months. Chance of infection over 2 months calculated for different levels of community transmission. See Table S4 for final assumptions.
Risk of dying from COVID-19	COVID-19 cases reported in Australia from January 2020 to 18/11/2021 were used to provide estimates of age-sex-specific case fatality rates. Data sourced from Australian NINDSS^24^. To convert reported age groups into those used in our model, calculations were based on age distribution of the Australian population^26^. See Table S5 for final assumptions.
Risk of getting (background) myocarditis	Multinational network cohort study from Australia, France, Germany, Japan, Netherlands, Spain, the UK and the USA reports background incidence of myocarditis and pericarditis per 100,000 person-years by age group and sex^27^. We assumed that 65% of reported myopericarditis cases were myocarditis, based on proportions from other studies that differentiate between them post-vaccination^28,29^. We converted incidence to probability of infection per person over 2 months. To convert reported age groups into those used in the model, calculations were based on age distribution of the Australian population^26^. See Table S6 for final assumptions.
Risk of dying from (background) myocarditis	Study reports incidence of fatal myocarditis in Finland per 100,000 person-years by age group and sex as total risk^30^, but not as case fatality rate. We converted incidence per 100,000 person-years to probability per person over 2 months (in the general population), then used these values for each age-sex subgroup as the numerator and the respective values for node ‘Risk of getting (background) myocarditis’ as the denominator to calculate case fatality rate. When converting reported age groups to the age groups used in our model, calculations were based on the age distribution of the Australian population^26^. See Table S6 for final assumptions.
Risk of getting Pfizer vaccine-associated myocarditis	Therapeutic Goods Administration (TGA) reports rates of myocarditis from the Pfizer vaccine per 100,000 doses in Australia, from all doses and second doses^31^. From this we calculated rates from first doses. At the time of writing, the only data available for the third dose in Australia cited four reports of likely myocarditis from a third dose of Pfizer up to 09/01/2022 with 3,651,855 third doses given nationally up to that date (with no breakdown of proportion of doses by brand). As this information is very limited, we assumed the same rate of vaccine-associated myocarditis as the second dose. This assumption was based on data from Israel reporting that rates of Pfizer vaccine-induced myocarditis from the third dose was higher than after the first dose but lower than after the second dose^32^. To provide a conservative estimate and avoid underestimating the potential risk of myocarditis after the third dose, we assumed the same rates as the second dose, i.e., the ‘worst-case scenario’. See Table S7 for final assumptions.
Risk of dying from Pfizer vaccine-associated myocarditis	Case fatality rate from mRNA vaccine-associated myocarditis has not been reported widely, in part due to very low numbers. Data from USA Centers for Disease Control and Prevention (CDC) Vaccine Adverse Event Reporting System (VAERS)^33^. Reported 1195 myocarditis cases after mRNA vaccination (dose number not specified) in those aged under 30 years, of which two likely died from myocarditis, giving a case fatality rate of 0.17% (2/1195). We assumed the same case fatality rate for Pfizer and other mRNA COVID-19 vaccines, and the same case fatality rate in those aged ≥30 years.
Risk of getting SARS-CoV-2 infection-induced myocarditis	Study reports that 5.0% of patients with COVID-19 developed new-onset myocarditis^34^ based on electronic medical records in TriNetX, a global federated health research network. Published data were insufficient to stratify by age and sex. Age-sex breakdown of the patient cohort with COVID-19 and related myocarditis cases were provided by the authors through personal communication. Data from the original patient cohort in the study were no longer available; the patient data provided through personal communication was from an updated cohort and showed a lower total prevalence of myocarditis (~2.3%). See Table S8 for final assumptions.
Risk of dying from SARS-CoV-2 infection-induced myocarditis	Study reports a six-month all-cause mortality of 3.9% in COVID-19 patients with myocarditis, assuming that deaths were attributable to myocarditis^34^. Published data were insufficient to stratify by age and sex. Age-sex breakdown of the myocarditis cases and deaths were provided by the authors through personal communication. Data provided through personal communication were based on electronic medical records in TriNetX, reported with patient counts ≥10 rounded up to 10 to safeguard protected healthcare data. The case fatality rate for age-sex subgroups with 10 deaths was thus assumed to be < 1.00%, with a value of 1.00% used in the model to assume the worst-case scenario. For males aged 12–19 and 20–29 years, there were zero deaths out of 152 and 661 cases of myocarditis, respectively. To avoid using a 0% case fatality rate in the model, we assumed that 12–19 and 20–29-year-old males had the same case fatality rate as 30–39-year-old males (1.00%). We believe this is a reasonable assumption because in females there was no significant difference in case fatality rate between ages 12–19 and 20–29 years and 30–39 years. See Table S8 for final assumptions.
Age distribution of population^a^	Distribution based on Australian Bureau of Statistics national population estimates from September 2021^26^. See Table S9 for final assumptions. Note age group 0–11 years was excluded from this version of the model because they were not yet eligible for vaccination in Australia at time of writing. This age group can be added into the model when vaccine coverage increases and data on vaccine effectiveness become available.
Sex distribution of population^a^	Assumed 50% male, 50% female.
Pfizer vaccine coverage in population^a^	Assumed 5% of population of ages ≥12 years had no doses, 5% had one dose only, 60% had two doses only, 30% had three doses. These approximations were based on vaccine coverage data from Australian Government Department of Health COVID-19 vaccination data on 3 Jan 2022^35^, and our estimates of how coverage will increase over the coming months.
Community transmission at x% over 2 months^a^	Chance of symptomatic infection (x%) over 2 months, based on different levels of community transmission. Priors set to even distribution between categories, assuming that community transmission level will be selected when using the CoRiCal tool or running public health-level scenario analyses. See explanation above under ‘Risk of symptomatic infection under current transmission and vaccination status’.

^a^Note that prior distributions do not affect results of scenario analysis but enables the model to provide population-level estimates. Assumptions can be changed as the situation evolves.

The BN includes four input nodes (orange) for use in scenario analyses: Pfizer vaccine dose and time since second dose (n1), age (n2), sex (n3) and intensity of community transmission (n4). Community transmission scenarios were presented as probability of infection over 2 months to enable comparison of vaccination risks versus benefits, as vaccine effectiveness is expected to decrease over time (modelled using 2-month intervals for time since second dose). Transmission scenarios were based on ATAGI definitions of low/medium/high risk^11^ (equivalent to 0.016%, 0.149%, 1.920% chance of infection over 2 months), and 1%, 2%, 5% and 10% chance of infection over 2 months. The model contains six intermediate nodes (yellow): Pfizer vaccine-associated myocarditis (n5), background incidence of myocarditis (n6), vaccine effectiveness (n7, n8), relative risk of symptomatic infection based on age and sex (n9), and incidence of COVID-19-related myocarditis (n11).

Two model versions were constructed employing distinct definitions of the ‘Pfizer vaccine dose and time since dose 2’ node (n1):

- Version 1: Pfizer vaccine doses defined as no doses, first dose, second dose and third dose. This version allows estimation of the probability of vaccine-associated myocarditis with each dose of vaccine, and was used in the coding of the CoRiCal online tool for providing individualised risk estimates.
- Version 2: Pfizer vaccine doses defined as no doses, received only one dose, received two doses and received three doses. This version allows estimation of the probability of deaths in the target population based on vaccine coverage rates, and is the model used henceforth for public health risk-benefit analyses.

### Model validation

All authors agreed that the final model accurately represented the variables, their states and associations within the model’s scope, in a manner consistent with the best current evidence. Manual calculations of risk estimates for multiple scenarios performed using the data sources and pre-defined assumptions matched the model outputs, validating the BN’s predictive behaviour (Supplementary Table 11).

### Risk-benefit analysis

The first risk-benefit analysis estimated the risks of background myocarditis, Pfizer vaccine-associated myocarditis and myocarditis in patients with symptomatic COVID-19. Based on background rates of myocarditis reported by Li et al.^27^ and Barda et al.^28^, our model estimated 2-month incidence of 10.0 (females aged 12–19 years) to 53.9 (males aged ≥70 years) cases per million, and overall case fatality rate (CFR) ranging from 1.2% to 4.3% for different age-sex subgroups (Supplementary Table 6).

Up to 09/12/2021 in Australia, age-sex-specific incidence of Pfizer vaccine-associated myocarditis cases ranged from zero to 24 per million after the first dose, and zero to 103 per million after the second dose (Supplementary Table 7), with no reported deaths. Our model assumed an overall CFR of 0.17% (two deaths out of 1195 cases) based on reports from the Centers for Disease Control and Prevention Vaccine Adverse Event Reporting System in the USA^33^ (Table 1).

**Fig. 2:**
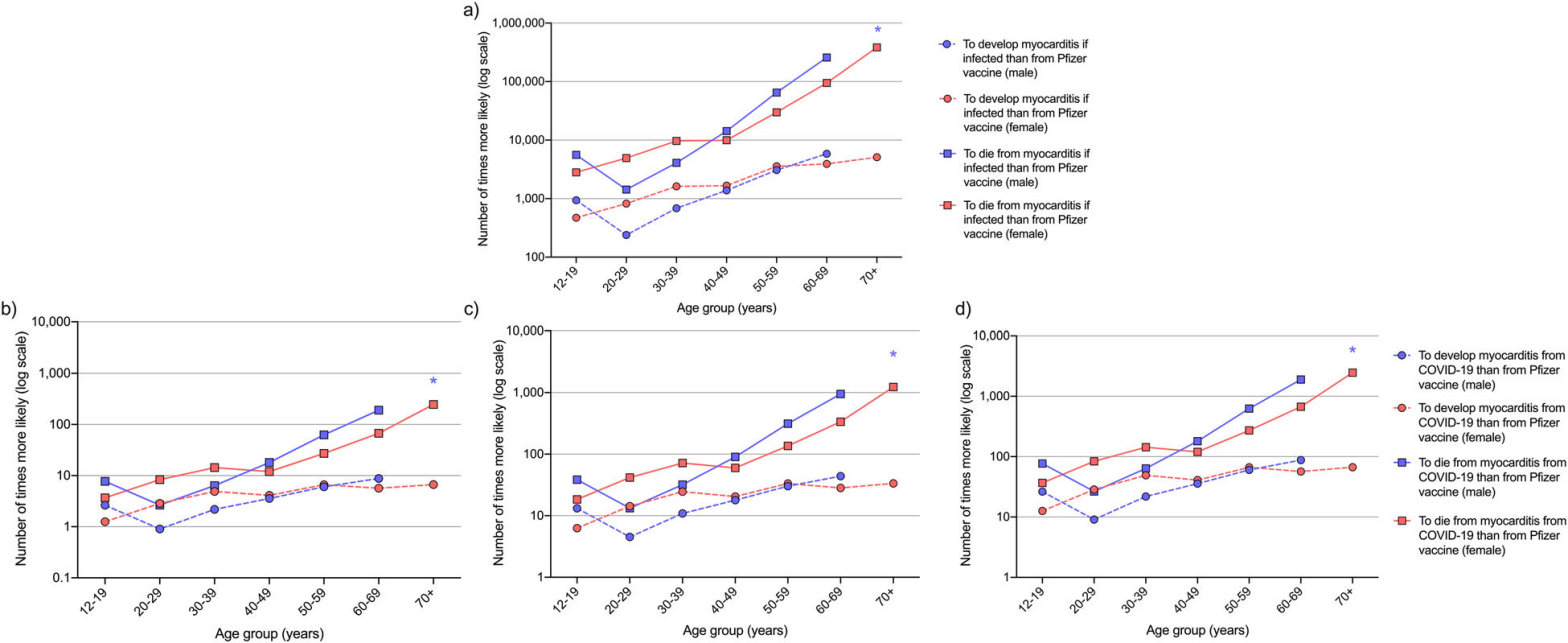
Comparison of the estimated risks of developing and dying from Pfizer vaccine-associated or COVID-19-related myocarditis. Number of times more likely (in log scale) for each age-sex subgroup to develop (circles) and die (squares) from myocarditis (**a**) in patients with symptomatic COVID-19 than from Pfizer vaccine-associated myocarditis. In those not yet infected with SARS-CoV-2, estimates for developing and dying from myocarditis over a 2-month period if 5% of population of ages ≥12 years had no doses, 5% had first dose, 60% had two doses (evenly distributed over 0 to < 2, 2 to < 4 and 4 to < 6 months since second dose) and 30% had three doses of Pfizer COVID-19 vaccine if community transmission equivalent to (**b**) 1%, (**c**) 5% and (**d**) 10% chance of infection over 2 months. *For males aged ≥70 years, Pfizer vaccine-associated myocarditis had an incidence of 0%. Note difference in y-axis scale between panel a and other panels.

At the time of writing, Australian data on myocarditis in COVID-19 patients were limited (Table 1). Model assumptions on the incidence and CFR of myocarditis in COVID-19 patients were obtained from an international cohort study by Buckley et al.^34^, and additional unpublished age-sex specific data from the study via personal communication with the lead author. Data showed incidence ranging from 1.66% to 13.74%, and CFR ranging from < 1% to 15.14%, depending on age and sex (Supplementary Table 8). Based on estimates from model version 2, Fig. 2 shows that, in a population aged ≥12 years, with vaccine coverage of 5% unvaccinated, 5% had one dose, 60% had two doses and 30% had three doses, the probability of developing myocarditis related to symptomatic COVID-19 once already infected with SARS-CoV-2 was 471 to 5847 times higher than developing Pfizer vaccine-associated myocarditis, depending on age group and sex (Fig. 2a, dashed lines). The probability of dying from myocarditis related to symptomatic COVID-19 was 1430 to 384,684 times higher than dying from vaccine-associated myocarditis, again depending on age group and sex (Fig. 2a, solid lines).

**Fig. 3:**
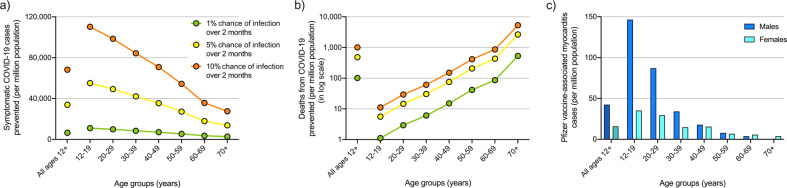
Comparison of estimated Pfizer vaccine-associated myocarditis cases to symptomatic COVID-19 cases and deaths prevented. Estimated COVID-19 cases (**a**) and deaths (**b**) (in log scale) prevented by age group over 2 months per million population if 5% of population of ages ≥12 years had no doses, 5% had first dose, 60% had two doses (evenly distributed over 0 to < 2, 2 to < 4 and 4 to < 6 months since second dose) and 30% had three doses of Pfizer COVID-19 vaccine if community transmission equivalent to 1% (green), 5% (yellow) and 10% (orange) chance of infection over 2 months. **c** Estimated cases of Pfizer COVID-19 vaccine-associated myocarditis over 2 months under the same vaccine coverage.

The second risk-benefit analysis performed estimated symptomatic COVID-19 cases and deaths prevented. Model version 2 was used to calculate expected symptomatic COVID-19 cases and deaths prevented over 2 months per million population aged ≥12 years, where 5% were unvaccinated, 5% had one dose, 60% had two doses (20% each with the last dose administered 0 to < 2, 2 to < 4 and 4 to < 6 months ago) and 30% had three doses. Figure 3a, b show the expected cases and deaths, respectively, prevented by age group under different community transmission intensities:

- 1% chance of symptomatic infection over 2 months (green), equivalent to average of 3645 cases per day in Australia (Supplementary Table 4);
- 5% chance of symptomatic infection over 2 months (yellow), equivalent to average of 7290 cases per day in Australia; and
- 10% chance of symptomatic infection over 2 months (orange), equivalent to average of 18,225 cases per day in Australia.

The model estimates that for a million 12–19 year-olds with this vaccine coverage, 11,029 symptomatic COVID-19 cases and one death would be expected to be prevented under 1% transmission (green) versus 110,288 cases and 11 deaths prevented under 10% transmission (orange) (Fig. 3a, b), with 146 expected cases of Pfizer vaccine-associated myocarditis in males and 35 cases in females (Fig. 3c). In contrast, for a million people aged ≥70 years, 2757 cases and 98 deaths would be expected to be prevented under the 1% transmission scenario, 27,566 cases and 981 deaths prevented under the 10% transmission scenario, with less than five expected vaccine-associated myocarditis cases in males or females. Calculations are detailed in Supplementary Table 12.

**Fig. 4:**
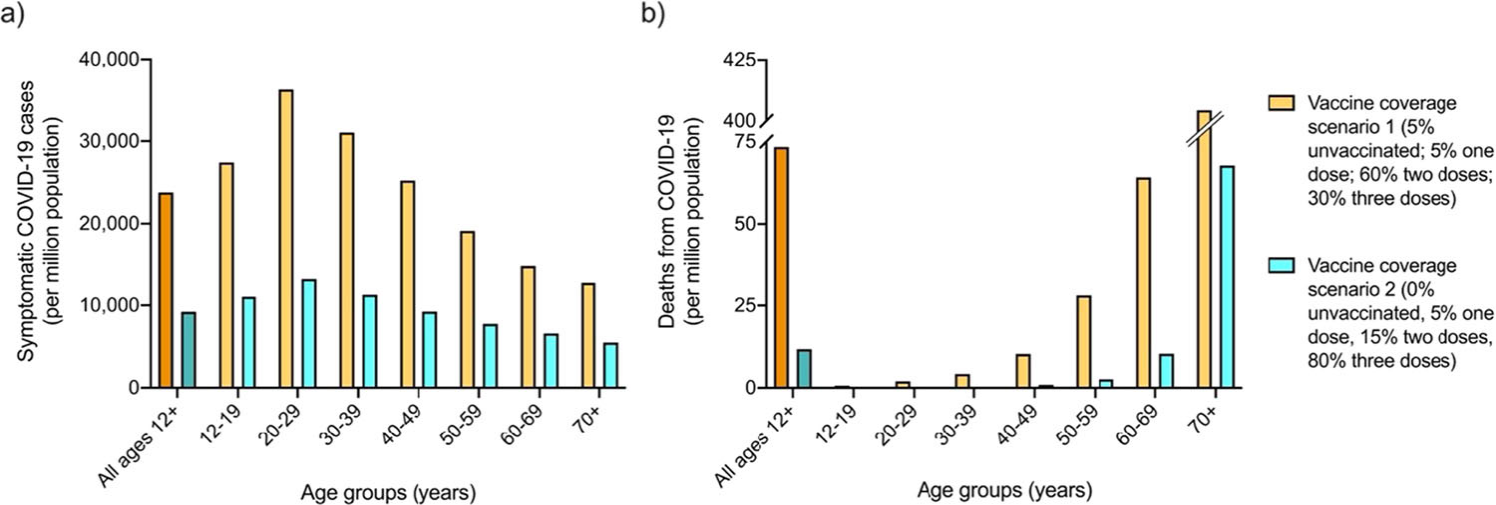
Estimated symptomatic COVID-19 cases and deaths under different vaccination coverage scenarios. Comparison of expected number of COVID-19 cases (**a**) and deaths (**b**) per million population by age groups under vaccine coverage scenario one (5% of population of ages ≥12 years had no doses, 5% had first dose, 60% had two doses [evenly distributed across time since second dose], and 30% had three doses of Pfizer COVID-19 vaccine), versus coverage scenario two (0% of population had no doses, 5% had one dose, 15% had two doses [evenly distributed across times since second dose] and 80% had three doses), under a transmission scenario equivalent to 10% chance of infection over 2 months.

The third risk-benefit analysis estimated symptomatic COVID-19 cases and deaths under different vaccination coverage scenarios. Model version 2 was further used to estimate expected symptomatic COVID-19 cases and deaths per million people if transmission intensity was equivalent to a 10% chance of infection over 2 months, if 5% were unvaccinated, 5% had one dose, 60% had two doses and 30% had three doses (scenario one) (Fig. 4, orange), versus if 0% of the population received no doses, 5% received the first dose only, 15% had two doses (5% each with the second dose administered 0 to < 2, 2 to < 4 and 4 to < 6 months ago), and 80% had three doses (scenario two) (Fig. 4, blue).

The model shows that for a million people aged 12–19 years with the vaccine coverage described in scenario one, 27,391 symptomatic COVID-19 cases and less than one death from COVID-19 would be expected under 10% transmission over 2 months, versus 11,042 cases and less than one death in scenario two. For one million people aged 20–29 years, 36,249 cases and two deaths could be expected in scenario one versus 13,168 cases and less than one death under scenario two. In contrast, for a million people aged ≥70 years, 12,694 cases and 404 deaths would be expected in scenario one versus 5487 cases and 68 deaths under scenario two.

### Sensitivity analysis

The first sensitivity analysis investigated the incidence of Pfizer vaccine-associated myocarditis. Therapeutic Goods Administration (TGA) reports between 14/10/2021 and 09/12/2021^36^ presented slight fluctuations in Pfizer vaccine-associated myocarditis incidence in Australia ranging from two to 37 cases per million depending on age-sex subgroup (Table 2). These small changes exerted no substantive impact on population-level estimates of the number of deaths. Model calculations also showed expected Pfizer vaccine-associated myocarditis deaths per million second doses to change only slightly during this time; differences ranged from 0.000 to 0.063 deaths per million by age-sex subgroup when comparing data from 14/10/2021 and 09/12/2021.

**Table 2:** Evolving evidence on incidence of Pfizer vaccine-associated myocarditis by age and sex in Australia in October–December 2021.

	Date	Sex	Age 12–19 years	Age 20–29 years	Age 30–39 years	Age 40–49 years	Age 50–59 years	Age 60–69 years	Age ≥70 years
Estimated incidence of myocarditis per million 2nd doses^a^	14/10/21	Male	75	22	6	10	3	0	0
		Female	14	12	3	10	3	0	0
	9/12/21	Male	103	59	15	11	1	0	0
		Female	25	19	6	9	4	0	0
Estimated deaths per million 2nd doses based on 0.34% CFR^b^	14/10/21	Male	0.128	0.037	0.01	0.017	0.005	0	0
		Female	0.024	0.02	0.005	0.017	0.005	0	0
	9/12/21	Male	0.175	0.1	0.026	0.019	0.002	0	0
		Female	0.043	0.032	0.01	0.015	0.007	0	0
Difference in estimated cases per million 2nd doses compared to 14/10/21	9/12/21	Male	28	37	9	1	2	0	0
		Female	11	7	3	1	1	0	0
Difference in estimated deaths per million 2nd doses compared to 14/10/21	9/12/21	Male	0.048	0.063	0.015	0.002	0.003	0	0
		Female	0.019	0.012	0.005	0.002	0.002	0	0

^a^Incidence of myocarditis in Australia reported by Therapeutic Goods Administration (TGA)^31^.

^b^CFR: Case fatality rate for all ages combined, calculated to be 0.17%, from ref. ^33^.

The second sensitivity analysis focused on vaccine effectiveness against developing and dying from symptomatic COVID-19. The model calculated that in a population where 5% are unvaccinated, 5% had one dose, 60% had two doses and 30% had three doses, a hypothetical 5% or 10% decrease in vaccine effectiveness against the delta variant would result in a 17.8% or 35.7% increase in estimated symptomatic cases, respectively, and a 23.9% or 54.7% increase in estimated expected deaths, respectively (Table 3). Thus, model estimates of cases and deaths are highly sensitive to reductions in vaccine effectiveness, necessitating frequent monitoring of and updating with emerging vaccine effectiveness data, particularly against new variants.

**Table 3:** Impact of theoretical reduction in vaccine effectiveness against delta variant on estimated deaths, assuming 5% of population of ages ≥12 years is unvaccinated, 5% had one dose, 60% had two doses and 30% had three doses.

		Current model assumptions	If 5% less effective	If 10% less effective
Average vaccine effectiveness for all ages ≥12 years against symptomatic infection after	1st dose (<3 weeks ago)	51.50%	46.50%	41.50%
	2nd dose (last dose 0 to < 2 months ago)	85.30%	80.30%	75.30%
	2nd dose (last dose 2 to < 4 months ago)	72.10%	67.10%	62.10%
	2nd dose (last dose 4 to < 6 months ago)	52.60%	47.60%	42.60%
	3rd dose (<4 months ago)	95.40%	90.40%	85.40%
% Increase in estimated symptomatic cases compared to current model assumptions of vaccine effectiveness		N/A	17.70%	35.40%
Average vaccine effectiveness for all ages ≥12 years against death after	1st dose (<3 weeks ago)	85.10%	80.10%	75.10%
	2nd dose (last dose 0 to < 2 months ago)	98.00%	93.00%	88.00%
	2nd dose (last dose 2 to < 4 months ago)	95.20%	90.20%	85.20%
	2nd dose (last dose 4 to < 6 months ago)	91.80%	86.80%	81.80%
	3rd dose (<4 months ago)	98.00%	93.00%	88.00%
% Increase in estimated deaths compared to current model assumptions of vaccine effectiveness		N/A	23.80%	54.90%

## Discussion

We developed a BN model to facilitate risk-benefit analysis of the Pfizer COVID-19 vaccine for the Australian population. Results from this model highlight the importance of both individual factors such as age, sex and vaccination status, and location-specific factors that reflect the current pandemic landscape, such as transmission intensity, case incidence and CFR from COVID-19, and COVID-19- and Pfizer vaccine-associated myocarditis. Our model could be used to help inform discussions and decision-making for population health managers, individuals and clinicians. In this way, the model may aid in policy development, public health management, increased public awareness and improved shared decision-making in medical consultations.

For Australians ≥12 years, we compared the risk of developing Pfizer vaccine-associated myocarditis, with the benefit of protection against developing and dying from symptomatic COVID-19 over 2 months under different transmission scenarios, if 5% were unvaccinated, 5% had a first dose 3 weeks ago, 60% had two doses (20% each with the last dose administered 0 to < 2, 2 to < 4 and 4 to < 6 months ago), and 30% had three doses with the last dose administered < 4 months ago. Overall, an Australian is 471 to 5847 times more likely to develop COVID-19-related myocarditis if infected with SARS-CoV-2 than vaccine-associated myocarditis, and 1430 to 384,684 times more likely to die from it, depending on age and sex (Fig. 2a). Under a transmission scenario where the chance of infection was 5%–10% over 2 months, an Australian who is not already infected with SARS-CoV-2 is 5–88 times more likely to develop myocarditis from COVID-19 than the Pfizer vaccine, and 13–2466 times more likely to die from it (Fig. 2c, d). Under a lower transmission scenario where the chance of infection was 1% over 2 months, all age-sex subgroups except for 20–29-year-old males are more likely to develop myocarditis from COVID-19 than the Pfizer vaccine, and all age-sex subgroups are more likely to die from it (Fig. 2b), an important finding for informing individual decision-making on Pfizer COVID-19 vaccination. Crucial also for informing public health decision-making, modelling showed that under any transmission level, younger age groups benefited the most from protection against symptomatic COVID-19 while older age groups benefited the most from protection against fatal COVID-19 (Fig. 3), both critical markers of vaccine effectiveness^37–39^. Younger age groups were at higher risk of developing vaccine-associated myocarditis than older groups, and males were at greater risk than females. We note that myocarditis was more common after COVID-19 compared to the background rates, especially in younger men. In comparison, vaccine-associated myocarditis also has a predilection for younger males but at a much lower prevalence than cases associated with symptomatic COVID-19. Importantly, in the main, vaccination is justified in all age groups because myocarditis is generally mild in the young^40–42^, and there is unequivocal evidence for reduced mortality in older individuals across all levels of community transmission.

While the above risk-benefit analyses were conducted assuming the Australian vaccine coverage at the time of writing, outcomes under other coverage rates can be assessed by the model to further inform public health decision-making. We compared the number of COVID-19 cases and deaths expected if the chance of infection was 10% over 2 months under a scenario where 5% are unvaccinated, 5% had a first dose 3 weeks ago, 60% had two doses (20% each with the last dose administered 0 to < 2, 2 to < 4 and 4 to < 6 months ago) and 30% had three doses with the last dose administered < 4 months ago, to those expected under a second scenario where 0% are unvaccinated, 5% had a first dose, 15% had two doses (5% each with the last dose administered 0 to < 2, 2 to < 4 and 4 to < 6 months ago) and 80% had three doses with the last dose administered < 4 months ago (Fig. 4). Younger age groups benefited from the steepest decline in expected case rates, with at least 23,000 fewer cases per million in 20–29 year olds. In contrast, older age groups benefited from the greatest decrease in expected deaths from COVID-19, with 337 fewer deaths per million expected in those aged ≥70 years.

The evidence on the risks versus benefits of COVID-19 vaccination was derived from an range of sources, each associated with some level of uncertainty. The use of probabilistic modelling techniques, such as BNs, can contribute somewhat to communicating this uncertainty by framing the outcomes in terms of probabilities. However, the nature of the evolving pandemic landscape means that the most update-to-date evidence will rarely be free of uncertainty. While we have not attempted here to quantify the uncertainty levels of the data used to parameterise individual components of the BN, all assumptions surrounding their use have been fully explained and justified. Sensitivity analysis was also used to examine the impact of some of the data uncertainties on the model estimates.

Sensitivity analysis showed model estimates to be robust against minor changes in the number of Pfizer vaccine-associated myocarditis cases (Table 2), but highly affected by changes in vaccine effectiveness against symptomatic infection and death (Table 3). At a public health level, this holds important implications for COVID-19 burden if new variants such as omicron, for which vaccine effectiveness is decreased, continue to emerge or if vaccine effectiveness proves to wane over time. While vaccine effectiveness would have to drop to a very low threshold for the associated myocarditis risk to outweigh the benefit of protection against symptomatic infection and death from COVID-19 in any age-sex-subgroup, this result highlights the importance of updating the model as new evidence becomes available, or new variants emerge.

Model estimates must be contextualised within the scope of the BN model, which does not currently consider comorbidities or personal behaviour that may influence an individual’s risks of acquiring COVID-19, their response to the infection, or their individual risk of myocarditis. Furthermore, limitations to the availability of Australian data introduces uncertainty in the model inputs, so results may change as more data become available. For example, the incidence of vaccine-associated myocarditis in Australia reported by TGA defines myocarditis based on varying degrees of certainty (e.g., confirmed diagnosis by clinical evidence, tests and imaging versus ECG versus possible diagnosis based on symptoms and a doctor’s report that myocarditis is the most likely diagnosis in the absence of medical tests and investigations)^31^. The rate also includes cases of myocarditis that occurred after vaccination but may not be vaccine-related (possibly due to background risk instead)^31^. This uncertainty in the diagnosis of myocarditis may also introduce reporting bias, e.g., self-reporting of adverse events may be influenced by exposure to relevant news in the media, resulting in temporary fluctuation in the reported incidence. In another example, at the time of writing, no Australian data were available on the incidence of Pfizer vaccine-associated myocarditis after the third dose and international data were deemed inappropriate as a substitute (see Table 1 assumptions), necessitating the use of rates for the second dose as a worst-case scenario. In a third example, when calculating the delta variant-specific CFR from COVID-19, ideally CFR for the unvaccinated population would be used, and the 2-to-3 week lag between diagnosis and death accounted for. This information was not available in Australia, so the assumptions were made that the time-window of a few months for the delta wave was long enough to minimise the effect of time lag from infection to death, and the great majority of deaths during the delta wave was in unvaccinated people. Other limitations arise from the model development process, where the use of expert elicitation may be perceived to introduce bias in the evidence viewed. This was minimised through broad literature searches and frequent meetings with external experts such as cardiologists about the quality of the data sources used in the model assumptions.

Despite these limitations, the use of an evidence-based BN to model the risks and benefits of COVID-19 vaccination has many advantages. BNs allow for interactive scenario analysis so the model was well-suited for use in programming CoRiCal, a free online tool aimed at better informing the public and helping clinicians to best advise patients on the risks and benefits of COVID-19 vaccination^19^. Another benefit of BNs is the ease of updating, allowing for future model updates to incorporate other outcomes such as long COVID, different patient groups such as those < 12 years and those with comorbidities, other vaccines such as Moderna, or different vaccine adverse events such as anaphylaxis. Finally, BNs are advantageous due to their integration of new data and different data sources in informing different aspects of the model. While this model has been designed for the Australian context, conditional probability tables (CPTs) can easily be re-populated wherever possible using data from another country.

In summary, we developed a BN to compare the risks and benefits of Pfizer COVID-19 vaccination in the Australian population, that incorporated emerging evidence of waning vaccine effectiveness over time and the recent recommendation of third doses, in order to assist clinicians with providing guidance about the Pfizer COVID-19 vaccine. In a community rather than individual context, the final model can also be used to calculate population-level estimates to help inform policy development and public health management. Although designed to compare risks of developing and dying from COVID-19, COVID-19- and Pfizer vaccine-associated myocarditis for the delta variant, the model can be updated to consider the omicron or other variants, other inputs such as patient comorbidities, and other outcomes such as long COVID.

## Methods

### Bayesian networks

**Fig. 5:**
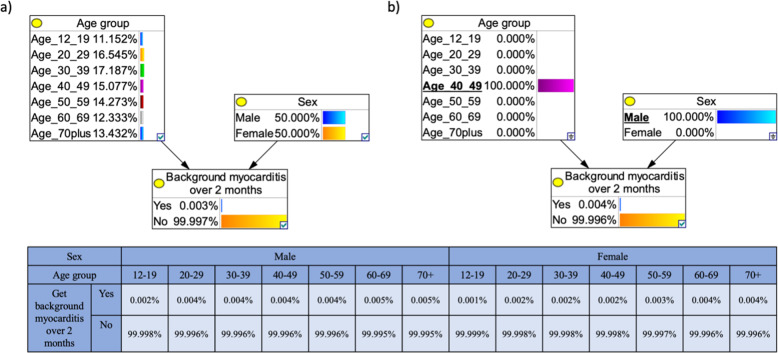
Example Bayesian network (BN) for modelling the risk of developing background myocarditis over 2 months based on age and sex. The output node, ‘Background myocarditis over 2 months’ is the child of two linked (arrow) parent nodes, ‘Age group’ and ‘Sex’. As these parent nodes do not have parent themselves, the probabilities of each of their possible states are determined by a prior distribution; the model adopts the age distribution of the Australian population and an even distribution of males and females. The conditional probability table for the outcome node ‘Background myocarditis over 2 months’, gives the probability for each state of this node dependent on the parent node states. **a** In the default state, the BN shows that the chance of developing background myocarditis (not from COVID-19 or the Pfizer vaccine) over 2 months is 0.003% (e.g., in a population of 100,000 people, we expect three to get myocarditis in a 2-month period). **b** An example of scenario analysis showing the chance of a 40–49-year-old male (underlined) developing background myocarditis over 2 months, the model calculates a 0.004% chance of myocarditis.

BNs are graphical displays of directional associations between variables, as defined by conditional probabilities^43^. Nodes represent variables and have multiple potential states (e.g., male and female), and associations are represented by arrows in the direction of parent (independent) to child (dependent) variable (Fig. 5). Probabilities are assigned to each potential node state via CPTs depending on parent node states or, in the case of no parents, prior distributions. The use of CPTs allows for integration of multiple data sources and formats including published figures, other literature and expert opinion, as well as easy updating when new data are presented^44^. BNs are also appropriate for analysing estimated or uncertain risks as they allow for sensitivity analysis to test multiple possible inputs^44^.

Throughout the COVID-19 pandemic, BNs have been used in decision-making^45^, risk assessment^46^ and analysis^47,48^. We have previously developed the first BN model for risk–benefit analysis of a COVID-19 vaccine, and used the model outputs to design an online tool to communicate the risks and benefits of the AstraZeneca COVID-19 vaccine in the Australian context^17–19^.

### Model design

The model was based on best evidence from multiple sources, designed through collaboration between subject matter experts (KRS, RP, JL, JES, CLL) including clinicians and researchers with expertise in virology, infectious disease epidemiology, cardiology, general practice and public health, and modellers (CLL, HJM, KM, JES), who facilitated the design process and generated the model. Subject matter experts identified relevant variables to be considered and their relationships, and agreed on reliable information sources for each variable. A conceptual model was drafted based on this and node states defined. The model was then evaluated and refined, with nodes and relationships being removed in the absence of reliable, quantitative evidence. The model focuses on ages ≥12 years due to insufficient data on younger age groups at the time of development.

### Myocarditis

Acute myocarditis can result in myocardial inflammation from either an infectious or immune-mediated aetiology^49^. Thus, our model compared the risk of Pfizer vaccine-associated myocarditis with the risk of myocarditis in COVID-19 patients. While often asymptomatic, myocarditis may present as chest pain, palpitations and/or dysrhythmias^49–51^ and can cause dilated cardiomyopathy, arrhythmia and/or sudden cardiac death^50,51^. In Australia, myocarditis is often diagnosed using electrocardiogram, serum troponin levels, inflammatory markers, chest X-ray, echocardiography and occasionally endomyocardial biopsy^12^. However, these methods can underestimate the presence of myocarditis in comparison to more sensitive cardiac magnetic resonance imaging (MRI), which is considered the gold-standard for non-invasive diagnosis worldwide^52,53^. The 2018 Lake Louise criteria for MRI-based diagnosis of myocarditis targets tissue-based imaging markers of oedema, hyperaemia, necrosis and fibrosis^54,55^. To ensure the diagnosis of myocarditis was made robustly in our model, data reporting myocarditis cases diagnosed via cardiac MRI were used wherever possible.

While both the Pfizer COVID-19 vaccine and COVID-19 itself may also be associated with pericarditis, either separately or simultaneously with myocarditis, this model focuses solely on myocarditis. This is because diagnostic criteria for pericarditis are not well-defined, and because it is less common than myocarditis. In studies that reported ‘myocarditis/pericarditis’, we estimated that ~65% of cases were attributable to myocarditis, based on proportions of cases reported in studies that differentiate between them^28,29^.

The definitions for vaccine-associated and infection-induced myocarditis used for the model reflect those used within the studies from which data were drawn. Vaccine-associated myocarditis was defined as confirmed or suspected myocarditis occurring within 2 months of vaccine administration (with most cases occurring within the first 10 days)^31^, and COVID-19-related myocarditis was defined as myocarditis that occurred within 6 months of COVID-19 diagnosis^34^.

### Data sources

CPTs were derived from data compiled by experts from published material, government reports and through dialogue with external clinical experts (e.g., cardiologists regarding the evidence for Pfizer vaccine-associated, COVID-19-related and background rate of myocarditis). Official Australian authority-issued data were employed whenever possible (e.g., national data on Pfizer vaccine-associated myocarditis). When this was unavailable, data were retrieved from other reliable and publicly available sources (e.g., background rates of myocarditis). Where Australian data were not readily available and international data were not suitable to use for the Australian context, expert opinion was sought. For example, there were limited data in Australia about Pfizer vaccine-associated myocarditis incidence and CFR after the third dose. While rates were reported in Israel and Singapore, these were deemed inappropriate to use in the model as reported rates from first and second doses in these countries were much lower than in Australia. However, both reported lower incidence of myocarditis after the third dose than the second dose. Therefore, to avoid underestimating the risk, the decision was made by the subject experts to use a conservative assumption that incidence after the third dose was the same as the second dose. For some variables, data analysis was required to obtain probabilities for the CPTs, e.g., converting COVID-19 case incidence into probability of infection over 2 months for the community transmission intensity node, or averaging data to fit the BN age categories. Table 1 and Supplementary Tables S1–9 summarise data sources, model assumptions and rationale.

The BN incorporates default prior distributions for age group (based on the Australian population’s age distribution), sex (50% male, 50% female) and vaccine coverage (5% of the population unvaccinated, 5% of received one dose, 60% received two doses [20% with the second dose administered 0 to < 2 months ago, 20% 2 to < 4 months ago and 20% 4 to < 6 months ago], and 30% received three doses [administered ~3 weeks ago]). Prior distributions do not influence scenario analyses results, e.g., once male sex is selected, outputs relate only to males regardless of the entered prior distribution of sexes. Prior distributions can also be altered to model-specific scenarios, e.g., different levels of vaccine coverage.

### Model validation

Subject experts and modellers reviewed the final model to evaluate if the network structure, variables, relationships, and assumptions adequately portrayed the current best evidence. Multiple scenarios were defined, and model outputs manually calculated from the data sources and pre-defined assumptions to validate the BN’s predictive behaviour (Supplementary Table 11).

### Risk-benefit analysis

We assessed the risks versus benefits of the Pfizer vaccine if 5% of the population received no doses, 5% received the first dose only, 60% had two doses (20% each with the last dose administered 0 to < 2, 2 to < 4 and 4 to < 6 months ago), and 30% had three doses within the last 2 months (third dose administered 4 to 6 months after second dose). We assumed the same vaccine coverage for all age groups. These priors were selected to represent predicted vaccination coverage at the time of writing. We compared the following risks (vaccine-associated myocarditis) and benefits (potential COVID-19 cases and deaths prevented) assuming the above vaccination coverage:

i. Estimated number of times more likely for a person with symptomatic COVID-19 to develop and die from COVID-19-related myocarditis, than for a person to develop and die from Pfizer vaccine-associated myocarditis.
ii. Estimated symptomatic COVID-19 cases and deaths prevented per million population if transmission intensity was equivalent to 1%, 5% or 10% chance of infection over 2 months, versus estimated cases of Pfizer vaccine-associated myocarditis.
iii. Estimated symptomatic COVID-19 cases and deaths per million if transmission intensity was equivalent to 10% chance of infection over 2 months, under the vaccination coverage scenario described above versus a possible future scenario where 0% of the population received no doses, 5% received the first dose only, 15% had two doses (5% each with the last dose administered 0 to < 2, 2 to < 4 and 4 to < 6 months ago), and 80% had three doses.

### Sensitivity analysis

Evidence informing many model inputs rapidly evolved throughout the model development process. We ran sensitivity analyses for two variables considered most likely to fluctuate over time, to evaluate the necessary frequency for updating model assumptions.

From October–December 2021, reported Pfizer vaccine-associated myocarditis incidence in Australia increased weekly but numbers remained very low. We assessed TGA reports from 14/10/2021 and 09/12/2021^36^ to evaluate how changes in data influenced model predictions of age-sex-specific myocarditis cases from the second vaccine dose, per million people. We also assessed model output sensitivity to hypothetical 5% and 10% decreases in vaccine effectiveness against both symptomatic infection and death for the delta variant.

### Reporting summary

Further information on research design is available in the Nature Research Reporting Summary linked to this article.

## Supplementary information

Supplementary Material

REPORTING SUMMARY

## Data Availability

The authors declare that all data supporting the findings of this study are available within the paper and its supplementary information files.

## References

[1] Swiss Agency for Therapeutic Products. *Swissmedic Grants Authorisation for the first COVID-19 vaccine in Switzerland* [accessed 22 January 2022], https://www.bag.admin.ch/bag/en/home/das-bag/aktuell/medienmitteilungen.msg-id-81761.html (2020).

[2] BioSpace. *Pfizer and BioNTech Expand Collaboration with U.S. to Provide 500 Million Additional COVID-19 Vaccine Doses at Not-for-profit Price for Donation to Poorest Countries* [accessed 22 January 2022], https://www.biospace.com/article/releases/pfizer-and-biontech-expand-collaboration-with-u-s-to-provide-500-million-additional-covid-19-vaccine-doses-at-not-for-profit-price-for-donation-to-poorest-countries/ (2021).

[3] COVID19 Vaccine Tracker. *Pfizer/BioNTech: Comirnaty* [accessed 22 January 2022], https://covid19.trackvaccines.org/vaccines/6/ (2022).

[4] Heller, J. *Israel Sees Probable Link between Pfizer Vaccine and Myocarditis Cases* (Reuters, 2021), https://www.reuters.com/world/middle-east/israel-sees-probable-link-between-pfizer-vaccine-small-number-myocarditis-cases-2021-06-01/.

[5] Centers for Disease Control and Prevention. *Clinical Considerations: Myocarditis and Pericarditis after Receipt of mRNA COVID-19 Vaccines among Adolescents and Young Adults* [accessed 22 January 2022], https://www.cdc.gov/vaccines/covid-19/clinical-considerations/myocarditis.html (2021).

[6] Wu, K. J. *Doctors Are Puzzled by Heart Inflammation in the Young and Vaccinated*. (The Atlantic, 2021). https://www.theatlantic.com/health/archive/2021/07/vaccination-myocarditis-kids/619339/.

[7] Melbourne Institute. *Vaccine Hesitancy Tracker* [accessed 22 January 2022], https://melbourneinstitute.unimelb.edu.au/publications/research-insights/ttpn/vaccination-report (2021).

[8] Australian Government Department of Health. *Who Can Get Vaccinated* [accessed 22 January 2022], https://www.health.gov.au/initiatives-and-programs/covid-19-vaccines/who-can-get-vaccinated#access-to-comirnaty-pfizer (2022).

[9] Wong MCS (2021). Acceptance of the COVID-19 vaccine based on the health belief model: a population-based survey in Hong Kong. Vaccine.

[10] Verger P, Peretti-Watel P (2021). Understanding the determinants of acceptance of COVID-19 vaccines: a challenge in a fast-moving situation. Lanc. Publ. Heal..

[11] Australian Technical Advisory Group on Immunisation. *Weighing up the potential benefits and risk of harm from COVID-19 vaccine AstraZeneca* [accessed December 2021], https://www.health.gov.au/sites/default/files/documents/2021/06/covid-19-vaccination-weighing-up-the-potential-benefits-against-risk-of-harm-from-covid-19-vaccine-astrazeneca_2.pdf (2021).

[12] Australian Government Department of Health. *Guidance on Myocarditis and Pericarditis after mRNA COVID-19 Vaccines* [accessed January 2022], https://www.health.gov.au/sites/default/files/documents/2021/10/covid-19-vaccination-guidance-on-myocarditis-and-pericarditis-after-mrna-covid-19-vaccines.pdf (2021).

[13] Australian Government Department of Health. *COVID-19 Vaccination–Vaccination Data–1 October 2021* [accessed January 2022], https://www.health.gov.au/resources/publications/covid-19-vaccination-vaccination-data-1-october-2021 (2021).

[14] Australian Government Department of Health. *ATAGI Statement on Revised Recommendations on the Use of COVID-10 Vaccine AstraZeneca*, 17 June 2021 [accessed January 2022], https://www.health.gov.au/news/atagi-statement-on-revised-recommendations-on-the-use-of-covid-19-vaccine-astrazeneca-17-june-2021 (2021).

[15] Tartof SY (2011). Effectiveness of mRNA BNT162b2 COVID-19 vaccine up to 6 months in a large integrated health system in the USA: a retrospective cohort study. Lancet.

[16] MacIntyre CR, Veness B, Berger D, Hamad N, Bari N (2021). Thrombosis with thrombocytopenia syndrome (TTS) following AstraZeneca ChAdOx1 nCoV-19 (AZD1222) COVID-19 vaccination—a risk-benefit analysis for people <60 years in Australia. Vaccine.

[17] Lau CL (2021). Risk-benefit analysis of the AstraZeneca COVID-19 vaccine in Australia using a Bayesian network modelling framework. Vaccine.

[18] Mayfield HJ (2022). Designing an evidence-based Bayesian network for estimating the risk versus benefits of AstraZeneca COVID-19 vaccine. Vaccine.

[19] Immunisation Coalition. *CoRiCal: Covid Risk Calculator* [accessed January 2022], https://corical.immunisationcoalition.org.au (2021).

[20] Chodick, G. et al. Assessment of effectiveness of 1 dose of BNT162b2 vaccine for SARS-CoV-2 infection 13 to 24 days after immunization. *JAMA Network Open 2021*. 10.1001/jamanetworkopen.2021.15985 (2021). 10.1001/jamanetworkopen.2021.15985 PMC8185600 34097044

[21] Perez, J. L. *Efficacy and Safety of BNT162b2 Booster–C4591031 2 Month Interim Analysis* [accessed December 2021], https://www.cdc.gov/vaccines/acip/meetings/downloads/slides-2021-11-19/02-COVID-Perez-508.pdf (2021).

[22] Nasreen S (2022). Effectiveness of COVID-19 vaccines against symptomatic SARS-CoV-2 infection and severe outcomes with variants of concern in Ontario. Nat Microbiol.

[23] Andrews N (2022). Duration of protection against mild and severe disease by COVID-19 vaccines. N. Engl. J. Med..

[24] Australian Government Department of Health. *Coronavirus (COVID-19) Case Numbers and Statistics—cases and Deaths by Age and Sex* [accessed December 2021], https://www.health.gov.au/news/health-alerts/novel-coronavirus-2019-ncov-health-alert/coronavirus-covid-19-case-numbers-and-statistics#covid19-summary-statistics (2021).

[25] Australian Government Department of Health. *Coronavirus Disease 2019 (COVID-19) Epidemiology Reports, Australia, 2020–2021* [accessed Dec 2021], https://www1.health.gov.au/internet/main/publishing.nsf/Content/novel_coronavirus_2019_ncov_weekly_epidemiology_reports_australia_2020.htm (2021).

[26] Australian Bureau of Statistics. *National, State and Territory population* [accessed December 2021], https://www.abs.gov.au/statistics/people/population/national-state-and-territory-population/mar-2021/31010do001_202103.xls (2021).

[27] Li X (2021). Characterising the background incidence rates of adverse events of special interest COVID-19 vaccines in eight countries: multinational network cohort study. BMJ.

[28] Barda N (2021). Safety of the BNT162b2 mRNA COVID-19 vaccine in a nationwide setting. N. Engl. J. Med..

[29] Su, J. R. *Myopericarditis following COVID-19 vaccination: updates from the Vaccine Adverse Event Reporting System (VAERS)* [accessed January 2022], https://www.cdc.gov/vaccines/acip/meetings/downloads/slides-2021-08-30/03-COVID-Su-508.pdf (2021).

[30] Kytö V, Saraste A, Voipio-Pulkki L, Saukko P (2007). Incidence of fatal myocarditis: a population-based study in Finland. Am. J. Epidemiol..

[31] Therapeutic Goods Administration. *COVID-19 vaccine weekly safety report—09-12-2021* [accessed December 2021], https://www.tga.gov.au/periodic/covid-19-vaccine-weekly-safety-report-09-12-2021 (2021).

[32] Vaccines and Related Biological Products Advisory Committee. *October 14–15, 2021 Meeting Presentation* [accessed December 2021], https://www.fda.gov/media/153086/download (2021).

[33] Oster M (2022). Myocarditis cases reported after mRNA-based COVID-19 vaccination in the US from December 2020 to August 2021. JAMA.

[34] Buckley BJR (2021). Prevalence and clinical outcomes of myocarditis and pericarditis in 718,365 COVID-19 patients. Eur. J. Clin. Investig..

[35] Australian Government Department of Health. *COVID-19 Vaccination–Vaccination data—3 January 2022* [accessed January 2022], https://www.health.gov.au/resources/publications/covid-19-vaccination-vaccination-data-3-january-2022 (2022).

[36] Therapeutic Goods Administration. *COVID-19 Vaccine Weekly Safety Report* [accessed December 2021], https://www.tga.gov.au/periodic/covid-19-vaccine-weekly-safety-report (2021).

[37] Sadarangani M (2021). Importance of COVID-19 vaccine efficacy in older age groups. Vaccine.

[38] Saadi, N. et al. Models of COVID-19 vaccine prioritization: a systematic literature search and narrative review. *BMC Med*. **19**, 10.1186/s12916-021-02190-3 (2021). 10.1186/s12916-021-02190-3 PMC8632563 34847950

[39] Moghadas SM (2021). The impact of vaccination on coronavirus disease 2019 (COVID-19) outbreaks in the United States. Clin. Infect. Dis..

[40] Montgomery J (2021). Myocarditis following immunization with mRNA COVID-19 vaccines in members of the US military. JAMA Cardiol..

[41] GOV.UK. *Coronavirus Vaccine–Weekly Summary of Yellow Card Reporting* [accessed January 2022], https://www.gov.uk/government/publications/coronavirus-covid-19-vaccine-adverse-reactions/coronavirus-vaccine-summary-of-yellow-card-reporting (2022).

[42] Mevorach D (2021). Myocarditis after BNT162b2 vaccine against COVID-19 in Israel. N. Engl. J. Med..

[43] Fenton, N. & Neil, M. Risk assessment and decision analysis with Bayesian networks. 2nd edn (CRC Press, 2019).

[44] Marcot BG (2017). Common quandaries and their practical solutions in Bayesian network modeling. Ecol. Model..

[45] Wang, J., Zhai, X. & Luo, Q. How COVID-19 impacts Chinese travelers’ mobility decision-making processes: a Bayesian network model. Information and Communication Technologies in Tourism. 557–563. 10.1007/978-3-030-65785-7_53 (2021).

[46] Fenton, N. E. et al. A Bayesian network model for personalized COVID-19 risk assessment and contact tracing. Preprint at 10.1101/2020.07.15.20154286v2 (2021).

[47] Prodhan, G. & Fenton, N. Extending the range of COVID-19 risk factors in a Bayesian network model for personalized risk assessment. Preprint at 10.1101/2020.10.20.20215814v1 (2020).

[48] Lai. K. & Yanushkevich, S. N. Machine reasoning to assess pandemics risks: case of USS Theodore Roosevelt. Preprint at https://arxiv.org/abs/2008.11040 (2020).

[49] Lampejo T, Durkin SM, Bhatt N, Guttmann O (2021). Acute myocarditis: aetiology, diagnosis and management. Clin. Med. J..

[50] Drory Y (1991). Sudden unexpected death in persons <40 years of age. Am. J. Cardiol..

[51] Pollack A, Kontorovich AR, Fuster V, Dec GW (2015). Viral myocarditis – diagnosis, treatment option, and current controversies. Nat. Rev. Cardiol..

[52] Luetkens JA (2017). Feature-tracking myocardial strain analysis in acute myocarditis: diagnostic value and association with myocardial oedema. Eur. Radio..

[53] Luetkens JS (2016). Comprehensive cardiac magnetic resonance for short-term follow-up in acute myocarditis. J. Am. Heart Assoc..

[54] Friedrich MG (2009). Cardiovascular magnetic resonance in myocarditis: a JACC White paper. J. Am. Coll. Cardiol..

[55] Ferreira VM (2018). Cardiovascular magnetic resonance in nonischemic myocardial inflammation: expert recommendations. J. Am. Coll. Cardiol..

