# Supplementary material for: A Bayesian network analysis quantifying risks versus benefits of the Pfizer COVID-19 vaccine in Australia
Source: NPJ Vaccines. 2022 Aug 11;7:93. doi: 10.1038/s41541-022-00517-6 (PMC9371378; doi:10.1038/s41541-022-00517-6)
Supplement: Supplementary file 1 — Supplementary Material [file 41541_2022_517_MOESM1_ESM.pdf]

Supplementary Materials

Supplementary Table 1. Pfizer COVID-19 vaccine effectiveness against symptomatic infection with SARS-CoV-2 delta variant, by age group.

Age (years)	1 dose[a]	2 doses (last dose 0 to <2 months ago)[b]	2 doses (last dose 2 to <4 months ago)[b]	2 doses (last dose 4 to <6 months ago)[b]	3 doses (<4 months post 3rd dose)[c]
12-19	53.1%	89.0%	79.5%	74.0%	96.5%
20-29	53.1%	86.5%	73.0%	48.0%	96.5%
30-39	53.1%	86.5%	73.0%	48.0%	96.5%
40-49	53.1%	86.3%	72.8%	51.8%	96.5%
50-59	53.1%	86.0%	72.5%	55.5%	95.1%
60-69	46.8%	82.8%	69.0%	50.8%	93.1%
≥70	46.8%	79.5%	65.5%	46.0%	93.1%

Sources:

[a]Chodick, G., Tene, L., Patalon, T., Gazit, S., Tov, A.B., Cohen, D., and Muhsen, K. (2021). Assessment of effectiveness of 1 dose of BNT162b2 vaccine for SARS-CoV-2 infection 13 to 24 days after immunization. *JAMA Network Open* 4(6):e2115985. https://doi.org/10.1001/jamanetworkopen.2021.15985. [1]

[b]Tartof, S.Y., Slezak, J.M., Fischer, H., Hong, V., Ackerson, B.K., Ranasinghe, O.N., et al. (2021). Effectiveness of mRNA BNT162b2 COVID-19 vaccine up to 6 months in a large integrated health system in the USA: a retrospective cohort study. *The Lancet* 398(10309):1407–1416. https://doi.org/10.1016/S0140-6736(21)02183-8. [2]

[c]Perez, J.L. (2021). Efficacy and safety of BNT162b2 booster – C4591031 2 month interim analysis. *Centers for Disease Control and Prevention*. Accessed 17 December 2021 from https://www.cdc.gov/vaccines/acip/meetings/downloads/slides-2021-11-19/02-COVID-Perez-508.pdf. [3]

Supplementary Table 2. Pfizer COVID-19 vaccine effectiveness against death if infected with SARS-CoV-2 delta variant, by age group.

Age (years)	1 dose[a]	2 doses (last dose 0 to <2 months ago)[b]	2 doses (last dose 2 to <4 months ago)[b]	2 doses (last dose 4 to <6 months ago)[b]	3 doses (<2 months post 3rd dose)[b]
12-59	89%	98.2%	95.3%	91.7%	98.2%
60-69	74%	97.6%	95.2%	92.0%	97.6%
≥70	74%	97.0%	95.2%	92.2%	97.0%

Sources:

[a]Nasreen, S., Chung, H., He, S., Brown, K.A., Gubbay, J.B., Buchan, S.A., et al. Effectiveness of mRNA and ChAdOx1 COVID-19 vaccines against symptomatic SARS-CoV-2 infection and severe outcomes with variants of concern in Ontario. Preprint at https://www.medrxiv.org/content/10.1101/2021.06.28.21259420v3 (2021). [4]

[b]Andrews, N., Tessier, E., Stowe, J., Gower, C., Kirsebom, F., Simmons, R., et al. (2021). Duration of protection against mild and severe disease by COVID-19 vaccines. *The New England Journal of Medicine.* https://doi.org/10.1056/NEJMoa2115481. [5]

Supplementary Table 3. Relative probability of infection by age group and sex for SARS-CoV-2 delta variant (chance of infection in each age-sex group if overall probability of infection of 1%).

Age (years)	Male	Female
0-11	1.37%	1.30%
12-19	1.41%	1.34%
20-29	1.41%	1.29%
30-39	1.18%	1.12%
40-49	0.98%	0.94%
50-59	0.76%	0.72%
60-69	0.51%	0.49%
≥70	0.39%	0.42%
**Overall**	**1.03%**	**0.97%**

Sources:

Australian Government Department of Health. (2021). Coronavirus (COVID-19) case numbers and statistics – cases and deaths by age and sex. *Australian Government Department of Health.* Accessed 17 December 2021 from https://www.health.gov.au/news/health-alerts/novel-coronavirus-2019-ncov-health-alert/coronavirus-covid-19-case-numbers-and-statistics#covid19-summary-statistics. [6]

Australian Government Department of Health. (2021). Coronavirus disease 2019 (COVID-19) epidemiology reports, Australia, 2020-2021. *Australian Government Department of Health.* Accessed 17 December 2021 from https://www1.health.gov.au/internet/main/publishing.nsf/Content/novel_coronavirus_2019_ncov_weekly_epidemiology_reports_australia_2020.htm. [7]

Supplementary Table 4. Probability of infection (over 2 months) based on different intensities of community transmission.

Intensity of community transmission	Cases per 100,000 over 16 weeks*	Cases per million over 2 months	Estimated % of population infected over 2 months	Equivalent to cases/day in Australia[a]
Zero	0	0	0.000%	0
Low*	29	157	0.016%	58
Medium*	275	1,490	0.149%	543
High*	3,544	19,197	1.920%	6998
1% chance of symptomatic infection over 2 months		10,000	1.000%	3645
2% chance of symptomatic infection over 2 months		20,000	2.000%	7290
5% chance of symptomatic infection over 2 months		50,000	5.000%	18,225
10% chance of symptomatic infection over 2 months		10,0000	10.000%	36,450

* Definitions of low, medium, and high transmission (cases per 100,000 over 16 weeks) as defined by [9]. Low: similar to first wave in Australia. Medium: similar to second wave in VIC. High: similar to Europe in January 2021.

[a]Based on Australian population of 21.87 million. [8]

Source:

Australian Technical Advisory Group on Immunisation. (2021). Weighing up the potential benefits and risk of harm from COVID-19 vaccine AstraZeneca. *Australian Government Department of Health.* Accessed 17 December 2021 from https://www.health.gov.au/sites/default/files/documents/2021/06/covid-19-vaccination-weighing-up-the-potential-benefits-against-risk-of-harm-from-covid-19-vaccine-astrazeneca_2.pdf. [9]

Supplementary Table 5. Cases, deaths, and case fatality rate of COVID-19 in Australia in ages ≥12 years by age and sex, 1/1/2020 to 18/11/2021.

	Male			Female		
Age (years)	Cases	Deaths	Case fatality rate	Cases	Deaths	Case fatality rate
12-19	11,934	1	0.01%	11,286	1	0.01%
20-29	20,066	6	0.03%	18,755	3	0.02%
30-39	17,324	12	0.07%	16,104	7	0.04%
40-49	12,277	28	0.23%	11,452	12	0.10%
50-59	9252	70	0.76%	8837	39	0.44%
60-69	5598	144	2.57%	5375	58	1.08%
≥70	5059	789	15.60%	5786	752	13.00%
**Total**	**81,510**	**1,050**	**1.29%**	**77,595**	**872**	**1.12%**

Sources:

Australian Government Department of Health. (2021). Coronavirus (COVID-19) case numbers and statistics – cases and deaths by age and sex. *Australian Government Department of Health.* Accessed 17 December 2021 from https://www.health.gov.au/news/health-alerts/novel-coronavirus-2019-ncov-health-alert/coronavirus-covid-19-case-numbers-and-statistics#covid19-summary-statistics. [6]

Australian Bureau of Statistics. (2021). National, state and territory population. *Australian Bureau of Statistics.* Accessed 15 December 2021 from https://www.abs.gov.au/statistics/people/population/national-state-and-territory-population/mar-2021/31010do002_202103.xls. [8]

Supplementary Table 6. Estimated background incidence and fatality of myocarditis over 2 months (in populations who have not received the Pfizer COVID-19 vaccine and have not been diagnosed with COVID-19).

	Incidence of myocarditis over 2 months (per million population)[b]		Incidence of fatal myocarditis over 2 months (per million population)[c]		Case fatality rates from myocarditis	
**Age (years)[a]**	**Male**	**Female**	**Male**	**Female**	**Male**	**Female**
12-19	19.0	10.0	0.3	0.3	1.3%	2.5%
20-29	40.1	17.3	0.5	0.3	1.2%	1.7%
30-39	40.1	20.6	0.9	0.4	2.3%	2.2%
40-49	40.1	23.8	1.3	0.7	3.3%	3.0%
50-59	44.4	28.7	1.1	0.9	2.6%	3.1%
60-69	50.9	35.8	1.3	1.0	2.5%	2.9%
≥70	53.9	39.6	1.6	1.7	3.0%	4.3%

Sources:

[a]Australian Bureau of Statistics. (2021). National, state and territory population. *Australian Bureau of Statistics.* Accessed 15 December 2021 from https://www.abs.gov.au/statistics/people/population/national-state-and-territory-population/mar-2021/31010do002_202103.xls. [8]

[b]Li, X., Ostropolets, A., Makadia, R., Shoaibi, A., Rao, G., Sena, A.G., et al. (2021). Characterising the background incidence rates of adverse events of special interest COVID-19 vaccines in eight countries: multinational network cohort study. *The BMJ* 2021(373):n1435. https://doi.org/10.1101/2021.03.25.21254315. [10]

[c]Kytö, V., Saraste, A., Voipio-Pulkki, L., and Saukko, P. (2007). Incidence of fatal myocarditis: a population-based study in Finland. American Journal of Epidemiology 165(5):570–574. https://doi.org/10.1093/aje/kwk076. [11]

Supplementary Table 7. Rates of myocarditis cases per million Pfizer COVID-19 vaccine doses in Australia by age and sex.

	First dose		Second dose		Third dose[a]	
**Age (years)**	**Male**	**Female**	**Male**	**Female**	**Male**	**Female**
12-19	24	6	103	25	103	25
20-29	17	7	59	19	59	19
30-39	17	8	15	6	15	6
40-49	5	5	11	9	11	9
50-59	7	2	1	4	1	4
60-69	4	6	0	0	0	0
≥70	0	4	0	0	0	0

[a]Assumed the same rates as after second dose because no data were available for rates after third dose.

Source:

Therapeutic Goods Administration. (2021). COVID-19 vaccine weekly safety report – 09-12-2021. *Australian Government Department of Health.* Accessed 17 December 2021 from https://www.tga.gov.au/periodic/covid-19-vaccine-weekly-safety-report-09-12-2021. [12]

Supplementary Table 8. COVID-19-related myocarditis cases, deaths, and case fatality rate in ages ≥12 years by age and sex, up to 6 months post-myocarditis diagnosis.

	Male					Female				
Age (years)	COVID-19 cases	Myocarditis cases	Incidence	Deaths	Case fatality	COVID-19 cases	Myocarditis cases	Incidence	Deaths	Case fatality
12-19	1106	152	13.74%	0[a]	<1.00%	12,291	204	1.66%	≤10[b]	<1.00%
20-29	31,758	661	2.08%	0[a]	<1.00%	54,404	1321	2.43%	≤10[b]	<1.00%
30-39	43,723	1025	2.34%	≤10[b]	<1.00%	76,988	1849	2.40%	≤10[b]	<1.00%
40-49	41,971	1044	2.49%	18	1.72%	65,273	1690	2.59%	≤10[b]	<1.00%
50-59	51,473	1242	2.41%	44	3.54%	68,627	1644	2.40%	23	1.40%
60-69	57,880	1286	2.22%	95	7.39%	65,223	1458	2.24%	59	4.05%
≥70	66,431	1314	1.98%	199	15.14%	74,800	1452	1.94%	183	12.60%
**Total**	**294,342**	**6724**	**2.28%**	**366**	**5.44%**	**417,606**	**9618**	**2.30%**	**305**	**3.17%**

[a]For males aged 12-19 and 20-29 years, there were zero deaths out of 152 and 661 cases of myocarditis, respectively. To avoid using a 0% case fatality rate in the model, we assumed that 12-19 and 20-29 year old males had the same case fatality rate as 30-39 year old males (1.00%).

[b]Patient counts of ≤10 were rounded up to 10 to safeguard protected healthcare data. Related case fatality rates were thus assumed to be <1.00%., with a value of 1.00% used in the model to assume the worst-case scenario.

Source:

Personal communication from authors regarding patient cohort described in: Buckley, B.J.R., et al. (2021). Prevalence and clinical outcomes of myocarditis and pericarditis in 718,365 COVID-19 patients. *European Journal of Clinical Investigation* 51(11):e13669. https://doi.org/10.1111/eci.13679. [13]

Supplementary Table 9. Age distribution of Australian population, March 2021.

Age (years)	Population	% of total population
0-11	3,828,247	14.90%
12-19	2,438,423	9.49%
20-29	3,617,689	14.08%
30-39	3,757,954	14.63%
40-49	3,296,519	12.83%
50-59	3,120,900	12.15%
60-69	2,696,731	10.50%
≥70	2,936,879	11.43%
**Total**	**25,693,342**	**100.00%**

Source:

[a]Australian Bureau of Statistics. (2021). National, state and territory population. *Australian Bureau of Statistics.* Accessed 15 December 2021 from https://www.abs.gov.au/statistics/people/population/national-state-and-territory-population/mar-2021/31010do002_202103.xls. [8]

Supplementary Table 10. Summary of nodes and relationships between nodes in a Bayesian network for assessing risks versus benefits of the Pfizer COVID-19 vaccine.

Node name (number)	Description	Potential values	Node type	Parent nodes	Child nodes
Pfizer vaccine dose & time since dose 2 (n1)	Vaccine dose number	None,<br>1st dose (<3 weeks ago),<br>2nd dose (last dose 0 to <2 months ago),<br>2nd dose (last dose 2 to <4 months ago),<br>2nd dose (last dose 4 to <6 months ago),<br>3rd dose	Input	Age group (n2)	n5, n7, n8
Age group (n2)	Age group (years)	12-19, 20-29,30-39, 40-49, 50-59, 60-69, ≥70	Input	N/A – Default priors: population distribution of Australia by age	n1, n5-9, n11, n13-15
Sex (n3)	Sex	Male, female	Input	N/A – Defaults to uniform distribution	n5, n6, n9, n11, n13-15
Community transmission at x% over 2 months (n4)	Probability of symptomatic infection over 2 months based on different levels of community transmission	None,<br>ATAGI[1] definitions of low, med, high, 1%, 2%, 5%, 10%	Input	N/A – Defaults set to uniform distribution	n10
Vaccine-associated myocarditis (n5)	Probability of developing myocarditis from the Pfizer COVID-19 vaccine	Yes, no	Intermediate	Pfizer vaccine dose & time since dose 2 (n1), Age group (n2), Sex (n3)	n12
Background myocarditis over 2 months (n6)	Probability of developing myocarditis over 2 months (background rate in those who have not had vaccine or infection)	Yes, no	Outcome	Age group (n2), Sex (n3)	n13
Vaccine effectiveness against symptomatic infection (n7)	Effectiveness of the vaccine at preventing symptomatic SARS-CoV-2 infection	Effective, ineffective	Intermediate	Pfizer vaccine dose & time since dose 2 (n1), Age group (n2)	n10
Vaccine effectiveness against death (n8)	Effectiveness of the vaccine at preventing deaths from symptomatic SARS-CoV-2 infection	Effective, ineffective	Intermediate	Pfizer vaccine dose & time since dose 2 (n1), Age group (n2)	n14
Relative risk of symptomatic infection by age and sex (n9)	Relative risk of symptomatic SARS-CoV-2 infection depending on age and sex	Yes, no	Intermediate	Age group (n2), Sex (n3)	n10
Risk of symptomatic infection under current transmission and vaccination status (n10)	Probability of symptomatic COVID-19	Yes, no	Intermediate	Community transmission at x% over 2 months (n4), Vaccine effectiveness against symptomatic infection (n7), Relative risk of symptomatic infection by age and sex (n9)	n11, n14
Myocarditis from COVID-19 (n11)	Probability of developing myocarditis related to SARS-CoV-2 infection	Yes, no	Intermediate	Age group (n2), Sex (n3), Risk of symptomatic infection under current transmission and vaccination status (n10)	n15
Die from vaccine-associated myocarditis (n12)	Probability of dying from COVID-19 vaccine-associated myocarditis	Yes, no	Outcome	Vaccine-associated myocarditis (n5)	N/A
Die from myocarditis (background) (n13)	Probability of dying from myocarditis (background rate in those who have not had	Yes, no	Outcome	Age group (n2), Sex (n3), Background myocarditis over 2 months (n6)	N/A

	COVID-19 vaccine or SARS-CoV-2 infection)				
Die from COVID-19 (n14)	Probability of dying from COVID-19	Yes, no	Outcome	Age group (n2), Sex (n3), Vaccine effectiveness against death (n8), Risk of symptomatic infection under current transmission and vaccination status (n10)	N/A
Die from COVID-19-related myocarditis (n15)	Probability of dying from COVID-19-related myocarditis	Yes, no	Outcome	Age group (n2), Sex (n3), Myocarditis from COVID-19 (n11)	N/A

[1]ATAGI: Australian Technical Advisory Group on Immunisation.

Supplementary Table 11. Results of manual calculations used to validate the mathematical assumptions used to parameterise the model. Values provided are from two independent modellers (blue, green) and model estimates (purple).

Questions		Calculated estimate
**1.** For a 30-39 year-old male, what is the chance of symptomatic infection under ATAGI high transmission if:	a) Not vaccinated	0.022701<br>0.022656<br>0.022656
	b) Had one dose (administered <3 weeks ago)	0.010647<br>0.010626<br>0.010626
	c) Had two doses (last dose 0-<2 months ago)	0.003065<br>0.003059<br>0.003059
	d) Had two doses (last dose 2-<4 months ago)	0.006129<br>0.006117<br>0.006117
	e) Had two doses (last dose 4-<6 months ago)	0.011804<br>0.011781<br>0.011781
	f) Had three doses	0.000795<br>0.000793<br>0.000793
**2.** For a 30-39 year-old male with symptomatic COVID-19, what is the chance of dying from COVID-19 if:	a) Not vaccinated	0.000693<br>0.000693<br>0.000693
	b) Had one dose (administered <3 weeks ago)	0.000076<br>0.000076<br>0.000076
	c) Had two doses (last dose 0-<2 months ago)	0.000012<br>0.000012<br>0.000012
	d) Had two doses (last dose 2-<4 months ago)	0.000033<br>0.000033<br>0.000033
	e) Had two doses (last dose 4-<6 months ago)	0.000057<br>0.000057<br>0.000057
	f) Had three doses	0.000012<br>0.000012<br>0.000012
*** 3.** Under ATAGI high transmission, if 5% of the population of ages ≥12 years have had no vaccine doses, 5% have had 1 dose only, 60% have had 2 doses only and 30% have had 3 doses, for one million 50-59 year-old females:	a) How many cases of vaccine-induced myocarditis would we expect?	6.700000<br>6.700000<br>6.700000
	b) How many vaccine-associated myocarditis-induced deaths would we expect?	0.022919<br>0.022919<br>0.022919
*** 4.** If 5% of the population of ages ≥12 years have had no vaccine doses, 5% have had 1 dose only, 60% have had 2 doses only and 30% have had 3 doses, for one million ≥70 year-old males:	a) How many symptomatic cases would we expect over 2 months if there was ATAGI medium transmission during this time?	183.0304<br>183.2208<br>183.2208
	b) How many deaths from COVID-19 would we expect over 2 months if there was ATAGI medium transmission during this time?	6.379250<br>6.389558<br>6.389558
**5.** If a 60-69 year-old female was diagnosed with COVID-19:	a) What are her chances of developing COVID-19-related myocarditis?	0.022354<br>0.022354<br>0.022354

	b) What are her chances of dying from COVID-19-related myocarditis (before diagnosis)?	0.000905<br>0.000905<br>0.000905
Additional population risk:	c) What are her background chances of developing myocarditis?	0.000036<br>0.000036<br>0.000036
	d) What are her background chances of dying from myocarditis?	0.000001<br>0.000001<br>0.000001

*Model estimates for Questions 3 and 4 are calculated using the population level model described in section 2.1, ‘Model description’.

Supplementary Table 12. Comparison of symptomatic COVID-19 cases prevented by Pfizer COVID-19 vaccine versus cases of Pfizer vaccine-associated myocarditis under different intensities of community transmission. Assuming transmission of delta variant; 5% of population of ages ≥12 years unvaccinated, 5% received first dose, 60% received two doses; 30% received three doses; vaccine effectiveness against symptomatic infection as reported in Table S1; age-sex-specific myocarditis incidence as shown in Table S7.

Age group (years)	Community transmission intensity (probability of infection over 2 months)	Estimated COVID-19 cases over 2 months (per million[a])		Estimated COVID-19 cases prevented over 2 months if 5% had 1st dose, 60% had 2 doses, 30% had three doses (per million[a])	Estimated cases of vaccine-associated myocarditis if 5% had 1st dose, 60% had 2 doses, 30% had 3 doses (per million[a])	Estimated cases of symptomatic COVID-19 prevented per vaccine-associated myocarditis cases
		0% vaccinated	5% had 1st dose, 60% had 2 doses, 30% had 3 doses			
**All ages ≥12[b]**	1%	9259	2378	6881	28	246
	5%	46,295	11,889	34,406		1229
	10%	92,590	23,777	68,813		2458
**12-19**	1%	13,768	2739	11,029	91	121
	5%	68,840	13,696	55,144		606
	10%	137,679	27,391	110,288		1212
**20-29**	1%	13,478	3625	9853	58	170
	5%	67,390	18,125	49,265		849
	10%	134,780	36,249	98,531		1699
**30-39**	1%	11,536	3103	8433	24	351
	5%	57,679	15,513	42,167		1757
	10%	115,259	31,026	84,333		3514
**40-49**	1%	9608	2522	7086	16	443
	5%	48,040	12,608	35,432		2215
	10%	96,081	25,216	70,864		4429
**50-59**	1%	7352	1912	5441	7	777
	5%	36,762	9559	27,203		3886
	10%	73,523	19,118	54,405		7772
**60-69**	1%	5045	1475	3570	5	714
	5%	25,224	7373	17,851		3570
	10%	50,448	14,746	35,702		7140
**≥70**	1%	4026	1269	2757	2	1379
	5%	20,130	6347	13,783		6892
	10%	40,260	12,694	27,566		13,783

[a]Per million population of each age group, or per million of all ages based on population distribution of Australia. [8]
[b]Calculations for all ages based on population distribution of Australia. [8]

References

1. Chodick, G., Tene, L., Patalon, T., Gazit, S., Tov, A.B., Cohen, D., and Muhsen, K. (2021). Assessment of effectiveness of 1 dose of BNT162b2 vaccine for SARS-CoV-2 infection 13 to 24 days after immunization. *JAMA Network Open* 4(6):e2115985. https://doi.org/10.1001/jamanetworkopen.2021.15985.
2. Tartof, S.Y., Slezak, J.M., Fischer, H., Hong, V., Ackerson, B.K., Ranasinghe, O.N., et al. (2021). Effectiveness of mRNA BNT162b2 COVID-19 vaccine up to 6 months in a large integrated health system in the USA: a retrospective cohort study. *The Lancet* 398(10309):1407–1416. https://doi.org/10.1016/S0140-6736(21)02183-8.
3. Perez, J.L. (2021). Efficacy and safety of BNT162b2 booster – C4591031 2 month interim analysis. *Centers for Disease Control and Prevention*. Accessed 17 December 2021 from https://www.cdc.gov/vaccines/acip/meetings/downloads/slides-2021-11-19/02-COVID-Perez-508.pdf.
4. Nasreen, S., Chung, H., He, S., Brown, K.A., Gubbay, J.B., Buchan, S.A., et al. Effectiveness of mRNA and ChAdOx1 COVID-19 vaccines against symptomatic SARS-CoV-2 infection and severe outcomes with variants of concern in Ontario. Preprint at https://www.medrxiv.org/content/10.1101/2021.06.28.21259420v3 (2021).
5. Andrews, N., Tessier, E., Stowe, J., Gower, C., Kirsebom, F., Simmons, R., et al. (2022). Duration of protection against mild and severe disease by COVID-19 vaccines. *The New England Journal of Medicine*. https://doi.org/10.1056/NEJMoa2115481.
6. Australian Government Department of Health. (2021). Coronavirus (COVID-19) case numbers and statistics – cases and deaths by age and sex. *Australian Government Department of Health.* Accessed 17 December 2021 from https://www.health.gov.au/news/health-alerts/novel-coronavirus-2019-ncov-health-alert/coronavirus-covid-19-case-numbers-and-statistics#covid19-summary-statistics.
7. Australian Government Department of Health. (2021). Coronavirus disease 2019 (COVID-19) epidemiology reports, Australia, 2020-2021. *Australian Government Department of Health.* Accessed 17 December 2021 from https://www1.health.gov.au/internet/main/publishing.nsf/Content/novel_coronavirus_2019_ncov_weekly_epidemiology_reports_australia_2020.htm.
8. Australian Bureau of Statistics. (2021). National, state and territory population. *Australian Bureau of Statistics.* Accessed 15 December 2021 from https://www.abs.gov.au/statistics/people/population/national-state-and-territory-population/mar-2021/31010do002_202103.xls.
9. Australian Technical Advisory Group on Immunisation. (2021). Weighing up the potential benefits and risk of harm from COVID-19 vaccine AstraZeneca. *Australian Government Department of Health.* Accessed 17 December 2021 from https://www.health.gov.au/sites/default/files/documents/2021/06/covid-19-vaccination-weighing-up-the-potential-benefits-against-risk-of-harm-from-covid-19-vaccine-astrazeneca_2.pdf.
10. Li, X., Ostropolets, A., Makadia, R., Shoaibi, A., Rao, G., Sena, A.G., et al. (2021). Characterising the background incidence rates of adverse events of special interest COVID-19 vaccines in eight countries: multinational network cohort study. *The BMJ* 2021(373):n1435. https://doi.org/10.1101/2021.03.25.21254315.
11. Kytö, V., Saraste, A., Voipio-Pulkki, L., and Saukko, P. (2007). Incidence of fatal myocarditis: a population-based study in Finland. *American Journal of Epidemiology* 165(5):570–574. https://doi.org/10.1093/aje/kwk076.
12. Therapeutic Goods Administration. (2021). COVID-19 vaccine weekly safety report – 09-12-2021. *Australian Government Department of Health.* Accessed 17 December 2021 from https://www.tga.gov.au/periodic/covid-19-vaccine-weekly-safety-report-09-12-2021.
13. Buckley, B.J.R., Harrison, S.L., Fazio-Eynullayeva, E., Underhill, P., Lane, D.A., and Lip, G.Y.H. (2021). Prevalence and clinical outcomes of myocarditis and pericarditis in 718,365 COVID-19 patients. *European Journal of Clinical Investigation* 51(11):e13669. https://doi.org/10.1111/eci.13679.
